# Evaluating the effectiveness of the geriatrician-designed “Older Patient Care and Seek Course” for family physicians

**DOI:** 10.3389/fmed.2026.1886875

**Published:** 2026-09-01

**Authors:** Esra Ates Bulut, Zeynep Kemik, Feride Gozen, Ali Ekrem Aydın, Ersin Akpinar, Derya Kaya, Ahmet Turan Isık

**Affiliations:** 1 Department of Geriatric Medicine, Adana City Training and Research Hospital, School of Medicine, University of Health Sciences, Adana, Türkiye; 2 Geriatric Science Association, Izmir, Türkiye; 3 Department of Geriatric Medicine, School of Medicine, Ondokuz Mayis University, Samsun, Türkiye; 4 Department of Family Physicians, Cukurova University, School of Medicine, Adana, Türkiye; 5 Department of Geriatric Medicine, Faculty of Medicine, Dokuz Eylul University, Izmir, Türkiye

**Keywords:** continuing education, continuing education evaluation, continuing medical education, general practitioners, geriatric medicine curriculum, geriatrics, healthcare education, primary care

## Abstract

**Background:**

The population is aging and many primary care providers report gaps in managing complex geriatric conditions. This study aims to evaluate the effectiveness of a six-hour training program delivered by geriatricians in improving family physicians' knowledge and understanding of geriatric syndromes.

**Methods:**

The study included 22 participants who attended an “Older Patient Care and Seek Course” held during an international family physicians congress. The course consisted of six hours of in-person, interactive presentations on the major geriatric syndromes. Prior to the training, participants completed an 18-item pre-test assessing their knowledge in areas such as preventive care, polypharmacy, pressure injuries, malnutrition, and cognitive impairment. Following the training, the same test was administered as a post-test to evaluate changes in knowledge. Each item was scored as one point.

**Results:**

Of the participants, 86.4% were women, with a median age of 30 years (range: 27–40). Additionally, 54.5% were family medicine specialists, and the remainder were family medicine residents. The median duration of post-graduate clinical experience was 5 years (range: 2–17). In terms of test results, the mean pre-test score was 9.00 ± 1.74, which significantly increased to 14.59 ± 1.89 on the post-test. The difference between pre- and post-test scores was statistically significant (*p* < 0.01).

**Conclusions:**

Education plays a critical role in recognizing and effectively addressing the needs of the geriatric population within primary healthcare settings. Implementing structured, interactive, comprehensive postgraduate CME programs led by geriatricians is an effective strategy to promote awareness and improve identification of geriatric syndromes among primary care specialists.

## 1 Introduction

The global demographic shift toward an aging population poses challenges for healthcare systems worldwide. By 2050, the proportion of individuals aged 60 and older is expected to double, reaching nearly 2.1 billion, with the most rapid growth occurring in low- and middle-income countries (1). This demographic transition makes it essential to have a primary care workforce proficient in geriatric medicine, capable of managing complex age-related conditions such as multimorbidity, malnutrition, frailty, cognitive impairment, and polypharmacy. However, studies consistently reveal significant gaps in geriatric knowledge and skills among family physicians, underscoring the urgent need for geriatric medicine training during family medicine residency programs and enhanced continuing medical education (CME) in this field (2). While the Accreditation Councils for Graduate Medical Education (ACGME) has established core geriatric competencies for internal and family medicine residents, similar standards for practicing family physicians are implemented inconsistently. Expanding geriatric CME for practicing family physicians, in partnership with geriatricians, is essential to bridge gaps in elderly care (3).

CME is crucial for the lifelong learning process of family physicians, allowing them to update their knowledge, refine clinical skills, improve decision-making, and adapt to evolving best practices in patient care. Professionally organized CME programs are vital to ensure that family physicians maintain high standards of practice, particularly in geriatrics, where specialized expertise is required for managing age-related comorbidities, polypharmacy, malnutrition, dementia, and preventive measures (4). Research shows that effective CME not only enhances medical knowledge but also improves diagnostic accuracy, reduces inappropriate prescribing, and enhances patient-centered communication (5).

There is a concern about the number of geriatricians or patient access in many countries (6). Given the shortage of geriatricians and the increasing burden of age-related diseases, there is a need for high-quality, standardized geriatric training programs. The effectiveness of CME relies not only on content but also on delivery methods, with interactive and case-based learning proving more impactful than passive lectures. Modern CME formats now include a variety of approaches, such as medical conferences, workshops, bedside teaching, and increasingly, online programs and digital learning platforms. This variety allows for tailored education that meets the specific needs of different practitioners (7).

To increase knowledge and awareness among family physicians, a six-hour in-person geriatric course was organized and delivered to family physicians at an international family medicine congress. The course covered key topics, including preventive measures such as vaccination and cancer screening for older adults, cognitive impairment, polypharmacy, malnutrition, and pressure injuries, all presented by a geriatric expert. To assess the program's effectiveness, an eighteen-question test was administered before and after the course. This study aimed to demonstrate the impact of a geriatric education program designed by expert geriatricians for primary care specialists, emphasizing the need for learner-centered approaches to enhance healthcare delivery for aging populations.

## 2 Materials and methods

In this study, the contribution of a one-day older patient care and seek course provided to family physicians was retrospectively investigated to assess its impact on raising awareness and knowledge levels about geriatric syndromes. Participant recruitment was initiated prior to the congress via an open call announced through the official congress platform. A total of 22 volunteer primary care physicians were enrolled. The educational intervention was conducted on May 10, 2024, as a structured 6-hour face-to-face course during the 23rd East Mediterranean Family Medicine Congress. The titles of the presentations were Approach to the Older Patient in Primary Care, Does Every Forgetfulness Indicate Dementia, Central Nervous System Drugs, Rational Prescribing and Polypharmacy, Nutritional Deficiency and Malnutrition in Older Adults, and Pressure Injury. These were specifically selected by the academic committee based on high-prevalence clinical challenges and the core competencies required for managing older adults in primary care settings. The primary learning goals were polypharmacy, rational prescribing, pressure injuries, malnutrition, diagnosis of cognitive impairment, comprehensive geriatric assessment, vaccination programs, and cancer screening tests. The small-group session was structured as interactive, case-based discussions and clinical practice examples consistent with current clinical guidelines. The educational program was organized by the Geriatric Science Association, and five expert geriatricians and a neurologist trained in geriatric medicine delivered the lectures. Each session was interactive, and questions were collected during the course delivery. A pre-test was administered to volunteer participants before the training to assess their knowledge of evaluating geriatric patients and recognizing geriatric syndromes (polypharmacy, malnutrition, pressure injuries, dementia, cancer screening, immunization, centrally active pharmacotherapy, cognitive impairment). The effectiveness of the training and the increase in awareness were evaluated using an 18-question pre- and post-test. The 5-option multiple-choice test items were constructed directly by the expert lecturers for each corresponding module, strictly adhering to the most recent clinical practice guidelines. Each correct answer received one point. The questionnaire covered various topics, including: two questions on cancer screening and vaccination recommendations for older patients; four questions on pressure injuries; three on malnutrition and enteral nutrition; two on cognitive impairment; two on central nervous system medications; and five on polypharmacy.

The “Exam Questions” are provided as a Supplementary File. Additionally, sociodemographic characteristics of the participants, such as age, gender, and professional experience, were recorded. Previous geriatric education was questioned as having received specialized postgraduate training in older adult health, clinical experience in a geriatrics department, or the opportunity to work alongside a geriatrician.

For statistical analysis, SPSS 25.0 was used. Categorical data was presented as numbers and percentages, while parametric numerical data was reported as mean and standard deviation. Non-parametric numerical data was expressed as median, minimum, and maximum values. The evaluation of increased awareness of geriatric patients and syndromes was based on the differences between pre- and post-test results. McNemar's test was used to assess the statistical difference in accuracy rates between the first and second tests, and the Wilcoxon signed-rank test was used to compare the pre- and post-test scores of the participants. A *p*-value < 0.05 was considered statistically significant.

## 3 Results

A total of twenty-two medical doctors attended the course, with 86.4% of them being women. The median age of the participants was 30 years, with ages ranging from 27 to 40 years. Additionally, 54.5% of the participants were family medicine specialists. The median post-graduate clinical experience among the participants was 5 years, with a range from 2 to 17 years. The demographic characteristics of the patients were shown in Table 1.

**Table 1:** Characteristics of participants.

Variables	*n* = 22
Age (median, min-max)	30 (27–40)
Sex (women, %)	86.4
Specialist (family physician, %)	54.5
Professional experience (median, min-max)	5 (2–17)
Geriatrics Education (%)	36.4

An analysis of the post-training accuracy rate revealed improvements in correct answers for 16 out of 18 questions. The increase in the correct-answer rate across the 10 questions following the course was statistically significant (*p* < 0.05). The post-training total scores were significantly higher than the pre-training scores (9.00 ± 1.74 vs. 14.59 ± 1.89, Z = −4.085, *p* < 0.01), with a very large effect size (r = 0.96). Two questions were answered correctly by all participants both before and after the course. Additionally, 5 of the 18 questions were answered correctly by all participants after the course. However, the incorrect response rate for one specific question actually increased after the course. The response rates of the participants were summarized in Table 2.

**Table 2:** The ratio of the correct answers to the questions.

Questions	Pretest (%)	Posttest (%)	*p*
1 Vaccination recommendations	4.5	68.2	**<0**.**01**
2 Cancer screening	4.5	77.3	**<0**.**01**
3 Presssure injury healing	68.2	100.0	**0**.**02**
4 Presssure injury healing	22.7	54.5	**0**.**04**
5 Presssure injury staging	45.5	95.5	**<0**.**01**
6 Wound dressings	22.7	81.8	**<0**.**01**
7 Malnutrition	31.8	86.4	**<0**.**01**
8 Nutritional evaluation	100.0	100.0	1.00
9 Enteral feeding	36.4	81.8	**<0**.**01**
10 Cognitive Impairment	77.3	50.0	**0**.**03**
11 Cognitive Impairment	50.0	68.2	0.29
12 Central Nervous System drugs	86.4	77.3	0.69
13 Central Nervous System drugs	50.0	68.2	0.22
14 Polypharmacy	40.9	100.0	**<0**.**01**
15 Prescription cascade	95.5	100.0	1.00
16 Aging physiologic changes	22.7	59.1	0.21
17 Rational prescribing	100.0	100.0	1.00
18 Polypharmacy	40.9	90.9	**<0**.**01**
Total Score (mean ± standard deviation)	9.00 ± 1.74	14.59 ± 1.89	**<0**.**01**

Bold values indicate statistical significance.

## 4 Discussion

The study reveals that CME courses can effectively update general practitioners and family medicine residents on important aspects of older patient care. In-person meetings, open conversations, and discussions with geriatric experts are particularly effective methods for achieving competency among family physicians.

A significant finding of the study was that the baseline accuracy rate for questions regarding vaccination and cancer screening among primary care specialists was only 4.5%, the lowest of all categories. After training, this accuracy rate improved dramatically to 70%. A recent study conducted in western Turkey included 984 older adults and reported that 45.6% of participants were vaccinated after age 65, with 35.3% receiving a vaccine within the preceding year. Among individuals, the influenza vaccine had the highest uptake at 41.3%, followed by pneumococcal (10.9%) and tetanus (5.5%) immunizations (8). Another study from Turkey examining vaccine awareness among physicians, mainly internists and family physicians, showed that only 20.9% of the participants correctly knew the routine vaccination recommendations for older adults (9).

Because of the lower vaccination ratios, it is crucial for family physicians to know and remind older patients of current vaccination recommendations. There were also noticeable improvements in correct responses related to pressure injury questions; however, the question regarding the classification of a fully healed pressure ulcer as a “mature and stable pressure ulcer” received the least correct answers after training. Pressure injury is a major geriatric syndrome which increase healthcare costs and poses challenge in home-based services, palliative care services and long-term care centers (10).

Participants consistently recognized, both before and after training, that reducing BMI below 20 kg/m² is not an appropriate strategy for lowering mortality in older patients. There was a notable improvement in knowledge regarding two malnutrition-related questions. Malnutrition screening is recommended for each older adult in any health setting (11), and it emphasizes increasing physicians' awareness to screen for and diagnose malnutrition (12). However, the accuracy in identifying the Apo *ε*4 allele as a risk factor for cognitive impairment decreased after training. Overall, correct response rates for this topic remained suboptimal, at 50%–60%, highlighting the need for specialized training in cognitive impairment, particularly in light of dementia screening recommendations for the high-risk older patients in primary care (13).

One key component of the undergraduate geriatric medicine curriculum is pharmacotherapeutics and deprescribing (14). For this purpose, major central nervous system pharmaceuticals, including their anticholinergic effects, were detailed in the course program. No significant improvement was observed for questions on serotonergic drugs and dopamine agonists (CNS medications), although post-training accuracy in this area was approximately 70%. The literature indicates that notable improvements in knowledge and prescribing skills were achieved following training for family physicians (15). Regarding the five questions on polypharmacy, participants answered four almost perfectly, and two questions showed statistically significant improvement. However, post-training accuracy for the question about which physiological aging change does not need to be considered when modifying medications remained at only 60%. The widespread incorrect responses to this question suggest that the question's clarity should be reconsidered.

Given the global increase in life expectancy and the medical complexity of older adults, all healthcare practitioners should receive foundational training in Geriatric Medicine during their undergraduate education. This training should focus on equipping students with the necessary knowledge, skills, and attitudes to effectively care for aging populations. Many countries are currently experiencing a shortage of geriatricians (16). In response, the European undergraduate curriculum in Geriatric Medicine was developed through a Delphi consensus process led by the European Union of Medical Specialists – Geriatric Medicine Section (UEMS-GMS) (14). The curriculum emphasizes key topics such as delirium, pharmacotherapeutics and deprescribing, healthy aging and health promotion, elder abuse, legal competencies, and telemedicine. Undergraduate programs that lack these components should be expanded or revised to align with evolving evidence and clinical needs. Internal medicine and family medicine residency programs—which produce the majority of adult care providers—play a crucial role in this continuum (17). However, only about half of graduating family medicine (48%) and internal medicine (52%) residents reported feeling well-prepared to manage older adult care (18).

As the older population continues to grow worldwide, it is critical that family physicians possess the expertise to manage complex and frail older patients (19). Interprofessional education (IPE) offers a promising approach to enhance collaboration and clarify professional roles within multidisciplinary teams. IPE involves interactive learning among students from two or more health or social care disciplines, aiming to foster high-quality, patient-centered care (20). Additionally, CME is also essential for practicing clinicians to maintain and improve their competency. Various teaching methods are used in geriatric CME programs. In addition to traditional lectures, active and interactive learning strategies—such as case-based learning and group discussions—have shown promise. Although direct comparisons of teaching methods and settings are limited (21), interaction remains a fundamental component of effective education. Online CME platforms that incorporate audio materials and discussion opportunities often result in higher participant satisfaction (22). The continuing education offering is subject to the goals of the organising institute, but even more to the needs and desires of the end user (4). Interactivity, the opportunity of online discussion and audio materials increase the satisfaction of professionals with the online course (23). Online courses must offer clear advantages, such as flexible scheduling, personalized learning paths, and alternative instructional approaches, to justify their use. The disadvantages—such as potential social isolation, technical difficulties, and reduced peer interaction—must be carefully weighed. Furthermore, online CME content should go beyond simple digitization of traditional materials; it should be dynamic, relevant, and case-based (24). Strategic development of CME programs requires identifying existing learning needs—both through physician self-assessment and institutional evaluation—setting measurable objectives and creating adaptable course structures. Integrated, learner-centered design is essential to ensure both the efficacy and efficiency of continuing education (7, 25). Compared with no intervention, CME programs—particularly those centered around educational meetings—modestly improve professional practice and, to a lesser extent, patient outcomes. Evidence suggests that educational meetings may be more effective than other behavioral interventions such as reminder systems or financial incentives in promoting adherence to clinical guidelines and best practices (25). A systematic literature review reported that the analysis of active-mode CME programs indicates that small-group, case-based methodologies and “train-the-trainer” models incorporating specialized toolkits were the most prevalent educational strategies. These interventions exhibited significant structural diversity, reflecting a highly adaptable approach to audience size and delivery format (26). Despite the challenges in collecting long-term outcome data, preliminary evidence suggests these frameworks successfully catalyze shifts in clinical behavior, foster local leadership, and enhance the instructional competencies of primary care physicians. The findings underscore that the most potent drivers of professional change are multimodal interventions that integrate practical resources with structured performance feedback and sustained dialogue between mentors and participants.

This study has several limitations. To start with, while the questions were developed with input from highly qualified experts in geriatric medicine, their quality and internal consistency were not formally validated. Additionally, the six-hour course may be insufficient to cover the breadth of geriatric syndromes such as delirium, sarcopenia, frailty, and falls. Furthermore, the training was conducted in an educational setting and lacked clinical assessment to evaluate its direct impact on patient care. The study's small sample size may limit the generalizability of the results. The single-group pre-test/post-test design without a control group also limits our ability to attribute the knowledge gain exclusively to the educational intervention. Finally, as a cross-sectional study, it does not allow for evaluation of long-term clinical outcomes. Future studies are necessary to validate the curriculum, enhance the quality of questions, and evaluate clinical outcomes such as prescribing appropriateness, hospital admissions, and healthcare utilization. Professional training programs on aging organized by non-governmental organizations often focus on a single topic. However, this training offers a comprehensive approach to caring for older patients and could serve as inspiration for programs that universities or ministries could develop.

## 5 Conclusions

Implementing structured, interactive, comprehensive postgraduate CME programs led by geriatricians is an effective strategy to promote awareness and improve identification of geriatric syndromes among primary care specialists.

## Data Availability

The raw data supporting the conclusions of this article will be made available by the authors, without undue reservation.

## References

[1] Decade of healthy ageing: baseline report. Geneva: World Health Organization (2020). Licence: CC BY-NC-SA 3.0 IGO

[2] Gazewood JD Vanderhoff B Ackermann R Cefalu C. Geriatrics in family practice residency education: an unmet challenge. Fam Med. (2003) 35(1):30–4. 12564861

[3] Besdine R Boult C Brangman S Coleman EA Fried LP Gerety M. Caring for older Americans: the future of geriatric medicine. J Am Geriatr Soc. (2005) 53(6 Suppl):S245–56. 10.1111/j.1532-5415.2005.53350.x 15963180

[4] VanNieuwenborg L Goossens M De Lepeleire J Schoenmakers B. Continuing medical education for general practitioners: a practice format. Postgrad Med J. (2016) 92(1086):217–22. 10.1136/postgradmedj-2015-133662 26850504 PMC4819632

[5] Thepwongsa I Kirby CN Schattner P Piterman L. Online continuing medical education (CME) for GPs: does it work? A systematic review. Aust Fam Physician. (2014) 43(10):717–21. 25286431

[6] Pitkälä KH Martin FC Maggi S Jyväkorpi SK Strandberg TE. Status of geriatrics in 22 countries. J Nutr Health Aging. (2018) 22(5):627–31. 10.1007/s12603-018-1023-7 29717764 PMC12876318

[7] Sherman L Hannu H Chappell K. An overview of continuing medical education/continuing professional development systems in Europe: a mixed methods assessment. J Contin Med Educ. (2024) 13(1):2435731. 10.1080/28338073.2024.2435731 PMC11619014 39640915

[8] Yalçın Gürsoy M Tanrıverdi G Özsezer G Chousko Mechmet F. Vaccination coverage and related factors among the elderly: a cross-sectional study from Turkey. Public Health Nurs. (2022) 39(2):390–7. 10.1111/phn.12972 34551144

[9] Ates Bulut E Badak SO Aksoy H Fadiloglu A Isik AT. The awareness and attitude of physicians to older adult routine vaccination scheme. Clin Interv Aging. (2022) 17:1581–8. 10.2147/CIA.S382311 36338873 PMC9635550

[10] Ates Bulut E Soysal P Isik AT. Frequency and coincidence of geriatric syndromes according to age groups: single-center experience in Turkey between 2013 and 2017. Clin Interv Aging. (2018) 13:1899–905. 10.2147/CIA.S180281 30323576 PMC6174888

[11] Volkert D Beck AM Cederholm T Cruz-Jentoft A Goisser S Hooper L. ESPEN Guideline on clinical nutrition and hydration in geriatrics. Clin Nutr. (2019) 38(1):10–47. 10.1016/j.clnu.2018.05.024 30005900

[12] Ohta R Nitta T Shimizu A Sano C. Role of family medicine physicians in providing nutrition support to older patients admitted to orthopedics departments: a grounded theory approach. BMC Prim Care. (2024) 25(1):121. 10.1186/s12875-024-02379-4 38641569 PMC11027398

[13] Siddiqui M Nyahoda T Traber C Elliott S Wang V Toledo-Franco L. Screening for cognitive impairment in primary care: rationale and tools. Mo Med. (2023) 120(6):431–9. 38144923 PMC10743330

[14] Masud T Ogliari G Lunt E Blundell A Gordon AL Roller-Wirnsberger R. A scoping review of the changing landscape of geriatric medicine in undergraduate medical education: curricula, topics and teaching methods. Eur Geriatr Med. (2022) 13(3):513–28. 10.1007/s41999-021-00595-0 34973151 PMC8720165

[15] Esmaily HM Savage C Vahidi R Amini A Dastgiri S Hult H. Does an outcome-based approach to continuing medical education improve physicians' Competences in rational prescribing? Med Teach. (2009) 31(11):e500–6. 10.3109/01421590902803096 19909027

[16] Flaherty E Bartels SJ. Addressing the community-based geriatric healthcare workforce shortage by leveraging the potential of interprofessional teams. J Am Geriatr Soc. (2019) 67(S2):S400–S8. 10.1111/jgs.15924 31074849

[17] Cheng HY Davis M. Geriatrics curricula for internal and family medicine residents: assessing study quality and learning outcomes. J Grad Med Educ. (2017) 9(1):33–45. 10.4300/JGME-D-16-00037.1 28261392 PMC5319626

[18] Blumenthal D Gokhale M Campbell EG Weissman JS. Preparedness for clinical practice: reports of graduating residents at academic health centers. JAMA. (2001) 286(9):1027–34. 10.1001/jama.286.9.1027 11559286

[19] Charles L Triscott JA Dobbs BM McKay R. Geriatric core competencies for family medicine curriculum and enhanced skills: care of elderly. Can Geriatr J. (2014) 17(2):53–62. 10.5770/cgj.17.95 24883163 PMC4038536

[20] Ploeg J Markle-Reid M Fisher A Bookey-Bassett S Chambers T Kennedy L. An exploration of experts' Perceptions on the use of interprofessional education to support collaborative practice in the care of community-living older adults. J Interprof Care. (2017) 31(5):638–47. 10.1080/13561820.2017.1347610 28792300

[21] Nguyen AL Duthie EA Denson KM Franco J Duthie EH. Positioning medical students for the geriatric imperative: using geriatrics to effectively teach medicine. Gerontol Geriatr Educ. (2013) 34(4):342–53. 10.1080/02701960.2013.809714 23972230

[22] Curran V Rourke L Snow P. A framework for enhancing continuing medical education for rural physicians: a summary of the literature. Med Teach. (2010) 32(11):e501–8. 10.3109/0142159X.2010.519065 21039092

[23] Parboosingh IJ Reed VA Caldwell Palmer J Bernstein HH. Enhancing practice improvement by facilitating practitioner interactivity: new roles for providers of continuing medical education. J Contin Educ Health Prof. (2011) 31(2):122–7. 10.1002/chp.20116 21671279

[24] Cook DA Garside S Levinson AJ Dupras DM Montori VM. What do we mean by web-based learning? A systematic review of the variability of interventions. Med Educ. (2010) 44(8):765–74. 10.1111/j.1365-2923.2010.03723.x 20633216

[25] Forsetlund L Bjørndal A Rashidian A Jamtvedt G O'Brien MA Wolf FM. Continuing education meetings and workshops: effects on professional practice and healthcare outcomes. Cochrane Database Syst Rev. (2021) 9(9):C003030. 10.1002/14651858.CD003030.pub2 PMC7138253 19370580

[26] Thomas DC Johnston B Dunn K Sullivan GM Brett B Matzko M. Continuing medical education, continuing professional development, and knowledge translation: improving care of older patients by practicing physicians. J Am Geriatr Soc. (2006) 54(10):1610–8. 10.1111/j.1532-5415.2006.00879.x 17038082

