# Supplementary material for: Evaluating the effectiveness of the geriatrician-designed “Older Patient Care and Seek Course” for family physicians
Source: Front Med (Lausanne). 2026 Sep 1;13:1886875. doi: 10.3389/fmed.2026.1886875 (PMC13574577; doi:10.3389/fmed.2026.1886875)
Supplement: Supplementary file 1 [file Table1.docx]

**Older Patient Care and Seek Course Questions**

**1st Question:** Which of the following statements about vaccinations in the old patient is incorrect?
a. The polysaccharide pneumococcal vaccine does not stimulate T cells and has a short memory.
b. The recombinant Varicella Zoster Virus vaccine is administered as a single dose.
c. Annual influenza vaccination is routinely recommended for those over 65.
d. If the first three vaccinations are completed for those over 65, tetanus vaccination should be administered every 10 years.
e. For individuals who received the polysaccharide vaccine before age 65, one dose of the 20-serotype conjugate pneumococcal vaccine is sufficient.

**2nd Question:** Which of the following statements about cancer screening in the old patient is incorrect?
a. Fecal occult blood tests measured with fecal immunochemical tests are influenced by food intake.
b. After age 75, decisions regarding breast cancer screening should be individualized.
c. Cervical cancer screening is discontinued for women over 65 with two previous negative HPV tests.
d. The effectiveness of prostate cancer screening has been demonstrated for ages 50-69.
e. Individuals over 65 with a 20-pack-year smoking history who quit 5 years ago should undergo annual low-dose chest CT scans.

**3rd Question:** In the physiological wound healing process when tissue integrity is disrupted, the correct chronological order of the phases is:
I-Tissue Remodeling Phase
II-Inflammation
III-Hemostasis
IV-Proliferation

a. II-III-I-IV
b. III-II-IV-I
c. III-IV-II-I
d. II-I-IV-III
e. III-I-II-IV

**4th Question:** A pressure ulcer that has completed its changes through the remodeling phase is defined as which type of pressure injury?
a. Closed pressure ulcer
b. Healed pressure ulcer
c. Mature and stable pressure ulcer
d. Open and healed pressure ulcer
e. Recurrent pressure ulcer

**5th Question:** The stage of pressure injury characterized by the presence of vesicles on the skin without epithelial loss is:
a. Stage 4
b. Stage 3
c. Stage 2
d. Stage 1
e. Unstageable

**6th Question:** Which of the following is correct about hydrocolloid wound dressings?
a. They can be used around the wound to prevent further skin trauma.
b. They are indicated for infected stage 2 pressure injuries.
c. They are the first choice for cavity wounds.
d. Their primary advantage is that they do not adhere to the wound bed.
e. They are contraindicated in diabetic foot ulcers.

**7th Question:** Which of the following statements about malnutrition in old patients is incorrect?
a. Routine screening is recommended to detect malnutrition in old patients.
b. Malnutrition increases the risk of sarcopenia, osteoporosis, falls, and fractures in old patients.
c. Weight loss is primarily used for diagnosis and follow-up.
d. A daily protein intake of 0.4-0.6 g/kg is recommended for maintaining muscle mass and body function in the elderly.
e. Enteral nutrition is indicated for patients consuming less than 50-60% of their nutritional needs orally for more than 7 days.

**8th Question:** Which of the following statements about nutritional disorders in the older adult is incorrect?
a. Anorexia of aging is a contributing factor to malnutrition.
b. Albumin and prealbumin are biochemical markers used to assess nutritional status.
c. Mini Nutritional Assessment (MNA) or NRS 2002 is recommended for evaluating the nutritional status of hospitalized old patients.
d. Dementia, depression, and polypharmacy increase the risk of malnutrition in geriatrics.
e. A body mass index (BMI) below 20 kg/m² is the target to reduce mortality in old patients.

**9th Question:** Which of the following statements about enteral nutrition is correct?
a. PEG should be planned for patients needing enteral nutrition for more than one week.
b. The first step in managing diarrhea in enterally-fed patients is to change the feeding formula.
c. Basal energy requirements are calculated using the Harris-Benedict formula.
d. In conditions like severe acute pancreatitis or ileus, the amount of enteral feeding may be reduced.

e. Semi-elemental enteral nutrition products may cause gastric intolerance and diarrhea.

**10th Question:** Which of the following is not a risk factor for cognitive decline?
a. Traumatic brain injury
b. Excessive alcohol consumption
c. Absence of apolipoprotein epsilon 4
d. Hearing impairment
e. Low social interaction

**11th Question:** Which of the following medications does not need to be questioned in a geriatric patient with complaints of forgetfulness?
a. Propranolol
b. Zolpidem
c. Quetiapine
d. Lansoprazole
e. Diphenhydramine

**12th Question:** Which of the following drug groups does not have serotonergic effects?
a. Opioids
b. Levodopa
c. Linezolid
d. Serotonin-norepinephrine reuptake inhibitors
e. Statins

**13th Question:** Which of the following statements about dopamine receptor agonists is incorrect?
a. They may lead to impulse control disorders.
b. Nausea and vomiting are common side effects.
c. Chronic use can lead to pretibial edema.
d. They are the first choice for parkinsonism symptoms in patients over 70.
e. They may cause psychotic symptoms such as hallucinations.

**14th Question:** What is the generally accepted definition of polypharmacy?
a. Daily use of five or more medications
b. Using multiple medications for chronic disease management
c. Using multiple medications to treat the same disease
d. Using two interacting medications
e. Using any medication without a rational indication

**15th Question:** Which of the following is incorrect about the prescription cascade?
a. It refers to treating a side effect caused by a medication as a new health issue with another medication.
b. Starting diuretic therapy for ankle edema caused by amlodipine is an example of a prescription cascade.
c. It is associated with adverse health outcomes and increased treatment costs.
d. Prescription cascades can always be prevented.
e. Taking a good medication history is essential to preventing prescription cascades.

**16th Question:** Which of the following physiological changes due to aging is not a primary consideration when adjusting medication in old patients?
a. Increased fibrosis in myocardial and autonomic tissue
b. Decreased ciliary activity in the respiratory system
c. Increased sclerotic nephrons in the kidney
d. Decreased lean muscle mass
e. Impairment of gastric mucosal protective barriers

**17th Question:** Which of the following is a physician-related risk factor for inappropriate medication use and polypharmacy in the aged?
a. Attending conferences and courses to maintain professional development in geriatrics
b. Despite the high patient load, include at least two older patients with polypharmacy in the deprescribing assessment every day.
c. Considering drug interaction alerts in e-prescribing systems when adjusting medications for old patients
d. Continuing a patient’s routine medications without questioning their indications to avoid conflict with the patient or family
e. Paying attention to safety warning letters from the Turkish Medicines and Medical Devices Agency

**18th Question:** An elderly woman with polypharmacy (taking candesartan, clopidogrel, metoprolol, atorvastatin, rabeprazole, prednisolone, donepezil, quetiapine, and oxybutynin) was diagnosed with uncomplicated urinary tract infection and started on oral ciprofloxacin. Which adverse effect is not expected to increase with fluoroquinolone use in this patient?
a. Arrhythmias due to prolonged QT interval
b. Tendinopathy (tendon rupture or tendinitis)
c. Aortic aneurysm and/or dissection
d. Peripheral neuropathy
e. Septic arthritis
